# Operationalizing interprofessional collaborative practice: an evolutionary concept analysis of collaborative team functioning in healthcare

**DOI:** 10.3389/fpsyg.2026.1898051

**Published:** 2026-10-26

**Authors:** Nora T. Kakati, Mollie F. Christiansen, Sean M. Reed

**Affiliations:** 1 Department of Medical Sciences, Khalifa University College of Medicine and Health Sciences, Abu Dhabi, United Arab Emirates; 2 College of Nursing, University of Colorado, Anschutz Medical Campus, Aurora, CO, United States; 3 Army Nurse Corps, College of Nursing, University of Colorado Anschutz Medical Campus, Aurora, CO, United States

**Keywords:** collaborative practice, concept analysis, healthcare teams, interprofessional collaboration, interprofessional collaborative practice (IPCP), team functioning

## Abstract

Interprofessional collaboration (IPC) describes professionals working together toward shared goals, however, it does not specify how that collaboration is enacted, observed, or evaluated in day-to-day practice. Interprofessional collaborative practice (IPCP) is often used interchangeably with IPC, yet the absence of a clear operational definition limits consistent implementation, evaluation, and its measurement development across healthcare teams. This concept analysis used Rodgers’evolutionary method to clarify IPCP and distinguish it from IPC by identifying the antecedents, attributes, consequences, and exemplars that characterize its enactment in practice. A total of 43 articles published between 2014 and 2025 were analyzed and organized using Tofthagen and Fagerstrøm’s three-phase framework. Antecedents included a collaborative practice-ready workforce, clearly defined roles, communication, trust, and a shared mental model. Attributes included shared decision-making, individualized care planning, mutual respect, and reciprocal relationships. Consequences included enhanced role awareness, team empowerment, improved care outcomes, and mutuality among team members, patients, and families. Structured Interdisciplinary Bedside Rounds emerged as a practical exemplar of IPCP, particularly when hierarchical barriers were reduced. Building on these findings, IPCP is defined as collaborative team functioning enacted through shared decision-making, reciprocal relationships, role clarity, and coordinated communication among healthcare professionals, patients, and families to achieve safe, effective, and person-centered care. Whereas IPC describes the intent and structure of working together, IPCP adds a focus on how collaboration is enacted, sustained, and evaluated in real-world teams. These findings offer a foundation for future work operationalizing, implementing, and evaluating collaborative team functioning across healthcare settings.

## 1 Introduction

Interprofessional collaboration among diverse health professionals is essential for delivering safe, effective, and person-centered care in increasingly complex healthcare systems (World Health Organization, 2010). The World Health Organization defines interprofessional collaboration (IPC) as a process in which two or more professionals work together to achieve shared goals and deliver high-quality care across settings (World Health Organization, 2010). This definition, and the broader IPC literature, establish who is involved in collaboration and why it matters: effective collaboration is consistently linked to improved patient outcomes and to shared responsibility, trust, and mutual respect within teams (Interprofessional Education Collaborative, 2023; World Health Organization, 2010). As healthcare systems continue to evolve, addressing multifaceted health challenges requires the coordinated efforts of interdisciplinary teams. Consequently, interprofessional collaborative practice (IPCP) remains a global priority for delivering safe, effective, person-centric care IPCP by fostering a team-based approach that actively involves healthcare professionals, patients, and families as contributors to care planning and decision-making (Interprofessional Education Collaborative, 2023; World Health Organization, 2010).

What remains less clear is how collaboration is enacted, observed, and evaluated in day-to-day practice. Interprofessional collaborative practice (IPCP) is often used alongside IPC, yet it lacks a clear operational definition that distinguishes the process of collaborating from its enactment in practice, which constrains the consistent implementation, evaluation, and measurement of IPCP across healthcare systems and interdisciplinary teams (Blakeney et al., 2021; Brashers et al., 2020). Existing scholarship often uses interprofessional collaborative practice, interprofessional collaboration, teamwork, interprofessional education, and interdisciplinary collaboration interchangeably, which contributes to conceptual ambiguity. This ambiguity also limits theory development and the consistent use of IPCP as a variable in empirical research.

Despite growing recognition of its importance, a substantial gap remains between IPCP as a valued ideal and IPCP as an operational construct that can be observed, implemented, and evaluated in practice. As healthcare teams continue to transition away from hierarchical, profession-centered models of care, team-based models in which every member’s expertise and voice are valued become increasingly important (MacMillan and Reeves, 2014). This transition is particularly difficult in acute care settings, where entrenched hierarchies and professional silos can undermine collaboration despite growing recognition of its importance (MacMillan and Reeves, 2014; Lingard et al., 2004). Effective IPCP extends beyond positive attitudes toward collaboration; it requires observable team-based behaviors, relational processes, shared decision-making, role clarity, coordinated communication, and meaningful engagement of patients and families (American Nurses Association, 2025; Brashers et al., 2020; MacMillan and Reeves, 2014; World Health Organization, 2010). Without greater conceptual clarity, IPCP may continue to be inferred from related concepts rather than examined as a distinct practice-based construct.

This evolutionary concept analysis addresses this gap using Rodgers’method, chosen for its alignment with the dynamic, context-dependent nature of IPCP and its capacity to clarify how a concept is used across health sciences (Rodgers and Knafl, 2000; Tofthagen and Fagerstrøm, 2010). The purpose of this analysis is to clarify IPCP as an operational construct for collaborative team functioning and to differentiate it from interprofessional collaboration more broadly. Specifically, this analysis aims to (1) identify the antecedents, attributes, and consequences that characterize how IPCP is enacted in practice, and (2) identify observable indicators that may support the future implementation and evaluation of IPCP across healthcare settings.

## 2 Theoretical framework

Interprofessional collaborative practice, which describes how healthcare team members function together, is fundamentally a team-level phenomenon whose enactment depends on disciplines within and beyond healthcare that specialize in the scientific study of group and team dynamics, including organizational psychology and organizational behavior (Golom and Schreck, 2018). Organizational psychology’s literature on team effectiveness treats this phenomenon as a core object of study, defining teams as individuals who interact toward common goals while holding interdependent roles and responsibilities embedded within a broader organizational system, using designated-position healthcare teams as an illustrative case (Mathieu et al., 2019) Effective interprofessional collaboration depends upon relational attunement, shared purpose, and the collective ability to engage in compassionate and responsive team processes (Interprofessional Education Collaborative, 2023; Wei and Watson, 2019). Within these collaborative spaces, psychological safety functions as a relational and moral condition that enables individuals to express concerns, seek clarification, acknowledge uncertainty, and participate authentically in collective decision-making without fear of negative repercussions (Edmondson and Bransby, 2023; O’Donovan et al., 2021). Psychological safety therefore functions as a core element of interprofessional collaborative practice, nurturing relational alignment and shared mental models that allow interprofessional teams to operate systematically.

This concept analysis accordingly stems from the basic tenets of psychological safety, a construct describing the interpersonal dynamics under which team members can engage in risk-taking, disclosure, and voice without fear of reprisal (Edmondson and Bransby, 2023; Edmondson and Lei, 2014; Edmondson, 1999). Building on Edmondson’s foundational work in organizational psychology (Edmondson, 1999; Edmondson and Bransby, 2023) have recently refined this construct, reconceptualizing psychological safety as a dynamic, context-sensitive, and highly subjective perception, continually shaped by trust, interactional history, and individuals’expectations regarding the interpersonal consequences of speaking up (Edmondson and Bransby, 2023). Their synthesis of empirical studies further establishes psychological safety as a mature, multidimensional construct spanning four interdependent domains, getting things done, learning behaviors, improving the work experience, and leadership, each directly relevant to how interprofessional teams function, learn, and sustain trust under interprofessional team complexity (Edmondson and Bransby, 2023).

This phenomenon extends beyond psychological safety into two further domains directly relevant to IPCP. Team effectiveness and team cognition research similarly identify leadership, mutual performance monitoring, backup behavior, adaptability, and team orientation as behavioral markers of effective teams (Salas et al., 2005), coordinated through shared mental models (Satcher et al., 2025), closed-loop communication, and mutual trust (Salas et al., 2005). The shared mental models construct is directly reflected in this analysis’ Shared Mental Model antecedent. Within healthcare specifically, this organizational lineage has been applied directly to interprofessional teams where psychological safety shapes knowledge-sharing behavior in clinical operations (Mura et al., 2016), enables engagement across professional and hierarchical boundaries within primary care teams (True et al., 2014), and its presence or absence has been identified as a key barrier or facilitator to interprofessional team functioning in primary care settings (Remtulla et al., 2021). These comprise findings consolidated in a systematic review of the organizational and relational factors that enable psychological safety across healthcare teams (O’donovan and Mcauliffe, 2020).

Caring Science and Convergent Care Theory are appropriate lenses for an explicitly interprofessional concept because neither is bound by professional identity in its own construction. Convergent Care Theory’s four processes include healthcare professional collaborative care as a distinct process defined by cross-professional coordination beyond nursing practice specifically, while Caring Science’s relational qualities, mutual respect, authentic presence, shared accountability, describe properties of relationship between any individual in a caring encounter, not properties exclusive to the nursing profession alone. While this analysis interprets the organizational and psychological literature above through this relational lens, the lens itself was selected for its applicability across professions, not despite it. This concept analysis is informed by a relational lens grounded in Caring Science and Convergent Care Theory (Wei, 2021; Wei et al., 2020), viewing this phenomenon as relevant within and across health professions, emphasizing IPCP as a relational practice through caring relationships, collaborative team functioning, and person-centric care. Caring Science frames collaboration as an ethical and relational commitment in which healthcare professionals engage with one another, patients, and families through mutual respect, authentic presence, and shared accountability (Wei and Watson, 2019). Convergent Care Theory extends this lens by describing how collaborative, person-centered care emerges through four interrelated processes: all-inclusive organizational care, healthcare professional collaborative care, person-centered precision care, and patient and provider self-care, achieved through competence, compassion, accountability, trust, sharing, and engagement (Wei, 2021).

Because nurses frequently coordinate care at the core of interprofessional communication, care transitions, patient advocacy, and shared decision-making across a vast combination of organizational, professional, patient, and family interfaces (American Nurses Association, 2025; Matziou et al., 2014), nursing literature served as an important focal, though non-exclusive, contributor to this analysis, while literature from other health professions was also included to preserve the inherently interprofessional nature of IPCP. This approach allowed the analysis to examine IPCP as a healthcare team concept while clarifying nursing’s contribution to its enactment in practice.

Read together, organizational psychology frameworks, psychological safety, Caring Science, and Convergent Care Theory frame IPCP as a dynamic, relational practice with an explicit ethical dimension: healthcare professionals are expected to practice collaborative care in ways that uphold dignity, shared values, and mutual accountability among team members, patients, and families in a safe IPCP care environment. This framework informs how the antecedents, attributes, and consequences of IPCP are interpreted throughout this analysis, particularly the emphasis on trust, mutual respect, and reciprocal relationships as mechanisms through which collaboration is enacted rather than simply intended.

## 3 Materials and methods

A comprehensive literature search was conducted to identify relevant English-language articles published between 2014 and 2024, with a targeted search update conducted in 2025 to capture the newly published evidence. Searches were performed in PubMed, Embase, and CINAHL using key terms such as interprofessional collaborative practice, interprofessional collaboration, interprofessional relations, teamwork, acute care, caring, and nursing. This search included empirical studies, systematic reviews, meta-analyses, concept analyses, and theoretical papers focused on IPCP within acute care settings. Full database search strings are provided in Appendix A.

The sample, as described in Rodgers’ method (Rodgers and Knafl, 2000), refers to the selected body of literature used for data collection based on predefined inclusion criteria. Articles were selected if they addressed interprofessional collaboration, interprofessional collaborative practice within healthcare teams including nursing, emphasized person-centered collaborative care delivery, were published in English, and were available in full text. Nursing literature served as an important contributor to the sample because nurses frequently function at the intersection of interprofessional communication, care coordination, patient advocacy, care transitions, and shared decision-making (American Nurses Association, 2025; Matziou et al., 2014). However, the sample was not limited to nursing literature. Studies from multiple healthcare professions were included where they addressed interprofessional collaboration to maintain the interprofessional scope of IPCP. Exclusion criteria included articles that were not available in full text, were published in languages other than English, or lacked relevance to IPCP, interprofessional collaboration, or collaborative team functioning.

The literature search yielded a total of 384 articles (PubMed = 197, Embase = 106, CINAHL = 81). Records were managed using EndNote 21 software, which was used to identify and remove 167 duplicate records, leaving 217 unique records for screening based on relevance and eligibility. A PRISMA-adapted approach was used to guide transparent reporting of the search, screening, and study selection process, summarized in the flow diagram in Figure 1 (Page et al., 2021). Screening proceeded in two stages. In the first stage, titles and abstracts were screened for relevance to interprofessional collaboration within healthcare teams, and 127 records that did not meet this criterion were excluded. In the second stage, the remaining 90 reports were retrieved in full text (none were unavailable) and assessed against the full inclusion and exclusion criteria. Of the 49 reports excluded following full-text assessment, 36 were excluded because nursing was not among the professions represented in the team or sample studied, 7 because they did not address IPCP or interprofessional collaboration as a central concept, 4 because they lacked relevance to collaborative team functioning, and 2 because the full-text articles were not available in English. This yielded 41 articles that met the inclusion criteria and were included in the primary analysis (Table 1). A targeted search update conducted in 2025, using the original search strategy and the same screening stages and eligibility criteria, resulting in a final sample of *n* = 43 articles included for analysis (Table 1). The search, screening, and selection process is summarized in Figure 1, adapted from the PRISMA 2020 flow diagram (Page et al., 2021).

**FIGURE 1:**
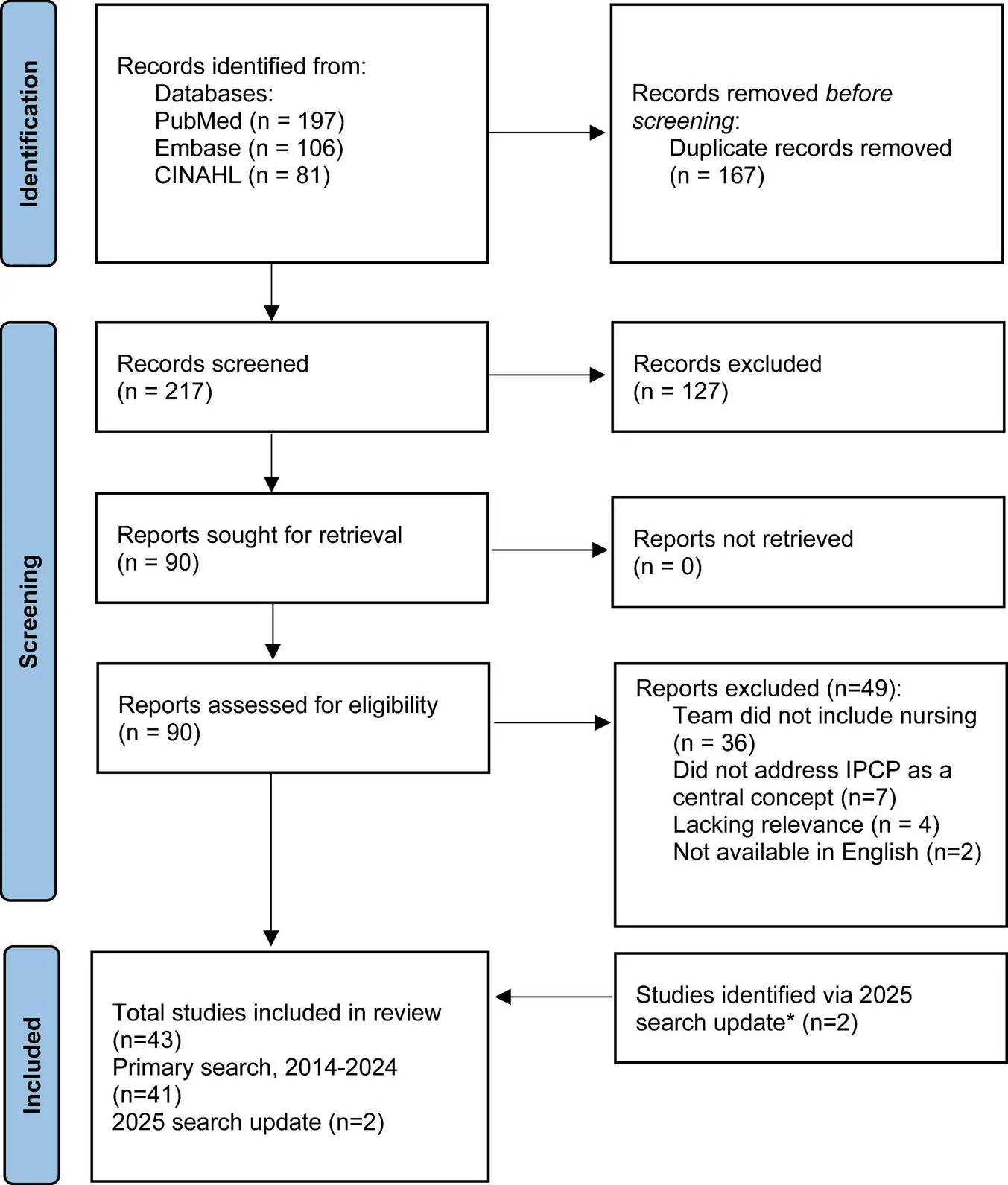
Integrative literature search diagram. Adapted from the PRISMA 2020 flow diagram (Page et al., 2021). The 2025 search update was run using the original search strategy with the same eligibility criteria.

**TABLE 1:** Literature matrix: interprofessional collaborative practice (primary search 2014–2024 with 2025 search update).

Item no.	Author, journal and year	Title	Type of data	Discipline	Country
1	Baggs, *American Journal of Critical Care*, 2022	Interprofessional, interdisciplinary, or multidisciplinary?	Letter	Interprofessional teams	USA
2	Bilodeau, *Journal of Interprofessional Care*, 2015	Interprofessional patient-centered practice in oncology teams: Utopia or reality?	Journal Article Qualitative Study	Interprofessional teams, nursing	Canada
3	Blakeney, *Journal of Interprofessional Education and Practice*, 2021	Measuring the impact of the national Train-the-Trainer Interprofessional Team Development Program (T3-ITDP) on the implementation of interprofessional education and interprofessional collaborative practice	Journal Article Quantitative Study	Interprofessional teams, nursing	USA
4	Bookey-Bassett, *Journal of Advanced Nursing*, 2017	Understanding interprofessional collaboration in the context of chronic disease management for older adults living in communities: a concept analysis	Journal Article Concept Analysis	Interprofessional teams, nursing	Canada
5	Brashers, *Journal of Interprofessional Care*, 2020	The ASPIRE model: grounding the IPEC core competencies for interprofessional collaborative practice within a foundational framework	Journal Article	Interprofessional Teams	USA
6	Brashers, *National Academies Press*, 2015	Measuring the impact of interprofessional education (IPE) on collaborative practice and patient outcomes	Book	Interprofessional teams	USA
7	Cao, *Critical Care Medicine*, 2018	Patient-centered structured interdisciplinary bedside rounds in the medical ICU	Journal Article Quantitative Study	Interprofessional teams, nursing	USA
8	Corbaz-Kurth, *Journal of Interprofessional Care*, 2026	How does trust emerge in interprofessional collaboration? A qualitative study of the significance, importance, and dynamics of trust in healthcare teams and networks	Journal Article Qualitative Study	Interprofessional teams, nursing	Switzerland
9	Cox, *Journal of Interprofessional Care*, 2016	Measuring the impact of interprofessional education on collaborative practice and patient outcomes	Editorial	Interprofessional teams	USA
10	Dahlke, *Canadian Journal on Aging / La Revue canadienne du vieillissement*, 2020	Perspectives about interprofessional collaboration and patient-centered care	Journal Article Mixed Methods	Interprofessional teams, nursing	Canada
11	Dolan, *Journal of Interprofessional Care*, 2025	Interprofessional socialization: a concept analysis	Journal Article Concept Analysis	Interprofessional teams, nursing	Canada
12	Fox, *Journal of Interprofessional Care*, 2015	Interprofessional collaborative patient-centered care: a critical exploration of two related discourses	Journal Article	Interprofessional teams, nursing	UK
13	Gausvik, *Journal of Multidisciplinary Healthcare*, 2015	Structured nursing communication on interdisciplinary acute care teams improves perceptions of safety, efficiency, understanding of care plan and teamwork as well as job satisfaction	Journal Article Mixed Methods	Interprofessional teams, nursing	USA
14	Gautama, *Journal of Holistic Nursing Science*, 2023	Collaboration between interprofessional education (IPE) and interprofessional collaborative practice (IPCP) in health education: a narrative review	Journal Article Review	Interprofessional teams, nursing	USA
15	Gilfoyle, *Pediatric Critical Care Medicine*, 2017	Improved clinical performance and teamwork of pediatric interprofessional resuscitation teams with a simulation-based educational intervention	Journal Article Quantitative Study	Interprofessional teams, nursing	Canada
16	Girard, *Human Resources for Health*, 2021	Interprofessional education and collaborative practice policies and law: an international review and reflective questions	Journal Article Review	Interprofessional teams	Canada
17	Green, *Journal of Chiropractic Education*, 2015	Interprofessional collaboration in research, education, and clinical practice: working together for a better future	Editorial	Interprofessional teams	USA
18	Haemmerli, *Journal of Multidisciplinary Healthcare*, 2021	Interprofessional collaboration in a new model of transitional care for families with preterm infants–the health care professional’s perspective	Journal Article Qualitative Study	Interprofessional teams, nursing	Switzerland
19	Interprofessional Education Collaborative, *IPEC*, 2023	Core competencies for interprofessional collaborative practice: 2023 update	Guideline	Interprofessional teams	USA
20	MacMillan, *Journal of Interprofessional Care*, 2014	Interprofessional education and collaboration: the need for a socio-historical framing	Journal Article Editorial	Interprofessional teams, nursing	USA
21	Matziou, *Journal of Interprofessional Care*, 2014	Physician and nursing perceptions concerning interprofessional communication and collaboration	Journal Article Quantitative Study	Interprofessional Teams, Nursing	Greece
22	McComb, *Journal of Advanced Nursing*, 2014	The concept of shared mental models in healthcare collaboration	Journal Article Concept Analysis	Interprofessional teams, nursing	USA
23	McGreal, *Nurse Education Today*, 2023	Nursing students’interprofessional socialization and readiness for interprofessional learning: a cross-sectional research study	Journal Article Quantitative Study	Interprofessional teams, nursing	Australia
24	Milton, *Scandinavian Journal of Trauma, Resuscitation and Emergency Medicine*, 2022	Healthcare professionals’ perceptions of interprofessional teamwork in the emergency department: a critical incident study	Journal Article Qualitative Study	Interprofessional teams, nursing	Sweden
25	Morgan, *International Journal of Nursing Studies*, 2015	Observation of interprofessional collaborative practice in primary care teams: an integrative literature review	Journal Article Integrative Review	Interprofessional teams, nursing	New Zealand
26	O’Donnell, *Systematic Reviews*, 2025	How can interprofessional collaboration be fostered and sustained in team-based care integration for older people in community settings? A realist evidence synthesis	Journal Article Systematic Review	Interprofessional teams	Ireland + International
27	Reeves, *Cochrane Database of Systematic Reviews*, 2017	Interprofessional collaboration to improve professional practice and healthcare outcomes	Journal Article Systematic Review	Interprofessional teams	International
28	Reinders, *Medical Education*, 2023	Interprofessional identity and motivation toward interprofessional collaboration	Journal Article Quantitative Study	Interprofessional teams	Netherlands
29	Schot, J*ournal of Interprofessional Care*, 2020	Working on working together. A systematic review on how healthcare professionals contribute to interprofessional collaboration	Journal Article Systematic Review	Interprofessional teams	Netherlands
30	Schwartz, *Journal of Interprofessional Care*, 2021	Structured interdisciplinary bedside rounds improve interprofessional communication and workplace efficiency among residents and nurses on an inpatient internal medicine unit	Journal Article Quasi-Experimental Study	Interprofessional teams, nursing	USA
31	Shirey, *Population Health Management*, 2021	Interprofessional collaborative practice model to advance population health	Journal Article	Interprofessional teams, nursing	USA
32	Sillero, *International Journal of Environmental Research and Public Health*, 2021	Enhancing interprofessional collaboration in perioperative setting from the qualitative perspectives of physicians and nurses	Journal Article Qualitative Study	Interprofessional teams, nursing	Spain
33	Stewart, *Shoulder & Elbow*, 2018	Stuck in the middle: the impact of collaborative interprofessional communication on patient expectations	Journal Article Editorial	Interprofessional teams	UK
34	Utley, *Human Kinetics*, 2020	Interprofessional education and collaboration: an evidence-based approach to optimizing health care	Book	Interprofessional teams	USA
35	Venville, *Journal of Interprofessional Care*, 2020	Building great health care teams: enhancing interprofessional work readiness skills, knowledge and values for undergraduate health care students	Journal Article Quantitative Study	Interprofessional teams, nursing	Australia
36	Verhaegh, Journal of Interprofessional Care, 2017	An exploratory study of healthcare professionals’ perceptions of interprofessional communication and collaboration	Journal Article Qualitative Study	Interprofessional teams, nursing	Netherlands
37	Ward, *BMC Medical Education*, 2017	Educating for interprofessional practice: moving from knowing to being, is it the final piece of the puzzle?	Journal Article Qualitative Study	Interprofessional teams, nursing	Australia
38	Wei, *International Journal of Nursing Sciences*, 2022	The development of an evidence-informed Convergent Care Theory: working together to achieve optimal health outcomes	Journal Article Theory Synthesis	Interprofessional Teams, Nursing	USA
39	Wei, *Journal of Interprofessional Care*, 2020	A culture of caring: the essence of healthcare interprofessional collaboration	Journal Article Qualitative Study	Interprofessional teams, nursing	USA
40	Wei, *Journal of Interprofessional Care*, 2022	A systematic meta-review of systematic reviews about interprofessional collaboration: facilitators, barriers, and outcomes	Journal Article Systematic Meta-Review	Interprofessional teams, nursing	International
41	Wei, *International Journal of Nursing Sciences*, 2019	Healthcare interprofessional team members’perspectives on human caring: a directed content analysis study	Journal Article Qualitative Study	Interprofessional teams, nursing	USA
42	Wood, *Journal of Interprofessional Care*, 2022	Interprofessional identity in clinicians: a scoping review	Journal Article Scoping Review	Interprofessional teams, nursing	Australia
43	Yamamoto, *Journal of Interprofessional Care*, 2022	Understanding interprofessional team delivery of patient-centered care: a qualitative secondary analysis	Journal Article Qualitative Study	Interprofessional teams	Canada

This concept analysis followed Rodgers’ evolutionary method (Rodgers, 1989; Rodgers and Knafl, 2000) using an integrative review approach. Literature was selected based on predefined eligibility criteria and conceptual relevance to IPCP. While the initial literature search, screening, and review was conducted by the first author (NK), inclusion and exclusion criteria were cross-checked by the second author (MC). The literature matrix (Supplementary Material 1) was shared with MC and discussed through virtual meetings, including discussion of included and excluded articles, with discrepancies resolved through feedback and discourse. To support consistent and transparent coding across reviewers, this literature matrix codebook was developed, capturing operational definitions and criteria for each antecedent, attribute, and consequence alongside the extracted themes, notes, and supporting quotes from each source article. Consistent with Rodgers’ evolutionary approach, these evolved iteratively while coding and reconciling source articles. This screening and reconciliation process took place within weekly Works-in-Progress sessions (WIPS), with methodological oversight provided by the third author (SR). Consistent with Rodgers’ evolutionary method (Rodgers and Knafl, 2000), formal quality appraisal was not performed because concept analysis prioritizes each source’s conceptual relevance over the methodological quality scoring more appropriate to intervention-focused systematic reviews.

This concept analysis followed Rodgers’ (2000) evolutionary method, structured through Tofthagen and Fagerstrøm’s (2010) three-phase, six-step framework. The (A) Initial Phase included: (1) choice of concept: determination of the concept of interest and its related terms and uses, including surrogate terms and related concepts; (2) context of the concept: determination and selection of the appropriate scope, setting, and sample; and (3) collection of relevant data specific to the scope and setting selected. The (B) Core Analysis Phase includes: (4) analysis of the data collected to determine defining definitions, surrogate terms, and related terms, including attributes, antecedents, and consequences within the selected context as extracted from the literature; and (5) determination of a reference and exemplar within the context of interest. The (C) Further Analysis Phase, the distinguishing feature of Rodgers’method, includes the final culminating activity, (6) further development of the concept through identification of hypotheses and future implications, reported in the Discussion (Foley and Davis, 2017; Rodgers, 2000; Tofthagen and Fagerstrøm, 2010). Consistent with this evolutionary approach, concept interpretation is understood to vary across disciplines, time, and context (Rodgers and Knafl, 2000). In this analysis, IPCP is examined within healthcare team functioning, with nursing as a focal, non-exclusive contributor to interprofessional practice.

## 4 Overview of the concept

### 4.1 Definitions

To date, a comprehensive definition of “interprofessional collaborative practice” as a standalone concept remains absent. While extensively studied, interprofessional collaboration is clarified through its core components: interprofessional, collaboration, and integration into practice. Interprofessional is defined as occurring between or involving two or more professions or professionals (Merriam-Webster, 2025a). Collaborative (adjective) stems from collaboration which is defined as to work jointly with others or together especially in an intellectual endeavor (Merriam-Webster, 2025b). Another definition is involving, or done by, several people or groups of people working together to create or produce something (Oxford, 2025a). Collaboration enables synergistic achievement collectively beyond individual contributions (Green and Johnson, 2015). Practice is defined as to do or perform, carry out or apply, and be professionally engaged in (an activity) (Merriam-Webster, 2025c), while also emphasizing action rather than abstraction (Oxford, 2025b). This distinction underscores IPCP as enacted practice rather than a conceptual ideal, highlighting the need for pragmatic implementation.

The overarching concept of interprofessional collaboration (noun) is well-established with clearly defined boundaries. The World Health Organization (WHO) defines it as a process in which two or more professionals work together to achieve shared goals and deliver the highest quality of care across settings (World Health Organization, 2010). While existing definitions emphasize collaboration among professionals, they provide limited guidance regarding how collaboration is enacted, observed, and evaluated in practice. Although the WHO definition identifies the participants involved in interprofessional collaborative practice, it does not specify the conditions, relational processes, observable behaviors, or outcomes that characterize effective implementation. This concept analysis extends existing definitions by conceptualizing IPCP as an operational and observable, construct supported by identifiable antecedents, attributes, consequences, and exemplars that may guide future implementation, evaluation, and measurement development within healthcare teams. IPCP therefore extends beyond collaborative intent by emphasizing how collaboration is enacted, sustained, and evaluated within real-world team interactions. This collaborative approach is recognized as a key strategy for addressing complex challenges in healthcare (Green and Johnson, 2015; Morgan et al., 2015; World Health Organization, 2010). Interprofessional collaboration supports person-centric care by prioritizing the needs of individuals, their families and communities, while simultaneously reorganizing them as integral components of the collaborative care team. Core elements include sharing, partnership, interdependency, and power dynamics (Green and Johnson, 2015). Morgan et al. (2015) propose a hierarchical relationship among these terms, positioning interprofessional collaboration as the broadest, umbrella concept, within which interprofessional collaborative practice describes how those principles are implemented in a specific practice setting, and teamwork denotes the deepest, most interdependent level of working together (Morgan et al., 2015).

Beyond establishing who collaborates and why, interprofessional collaboration is characterized in the literature by sharing, partnership, interdependency, and power dynamics among team members (Green and Johnson, 2015), and supports person-centered care by positioning patients, families, and communities as integral members of the care team (Bilodeau et al., 2015; Green and Johnson, 2015; World Health Organization, 2010). Building on this foundation, the present analysis focuses specifically on how collaboration characterized by these core elements is enacted, sustained, and evaluated in practice, an emphasis reflected throughout the antecedents, attributes, consequences, and exemplars presented below. In doing so, this analysis advances IPCP as an operational and observable form of collaborative team functioning that can support future empirical testing and measurement development.

### 4.2 Surrogate terms

Rodgers and Knafl (2000) define surrogate terms as alternative words or phrases used to represent a concept in different contexts, revealing terminological variations across disciplines and deepening conceptual understanding. Surrogate terms convey the essence of a concept using language other than the primary term selected by the researcher (Rodgers and Knafl, 2000; Tofthagen and Fagerstrøm, 2010). In the context of IPCP, commonly used surrogate terms include interprofessional collaboration (IPC), interprofessional education and collaborative practice (IPECP), teamwork, and collaboration. Notably, the terms interprofessional collaboration and collaborative practice are often used interchangeably in the literature, contributing to conceptual ambiguity. IPCP is frequently conflated with related terms such as interprofessional education (IPE) and IPECP, as well as with interdisciplinary and multidisciplinary collaboration, without clear distinctions involving parallel rather than integrated practice (Baggs, 2022; Blakeney et al., 2021; Bookey-Bassett et al., 2017; Brashers et al., 2020; Reeves et al., 2017; Zwarenstein et al., 2009). Similarly, a recent realist synthesis of interprofessional collaboration reported multiple, non-equivalent definitions and understandings of collaboration in circulation, reinforcing that conceptual fragmentation extends across the broader interprofessional and integrated-care literature (Bookey-Bassett et al., 2017; O’Donnell et al., 2025). Interprofessional is used throughout this analysis to reflect the focal concept. The term interdisciplinary is retained only when referring to named interventions, frameworks, or terminology used in the original sources.

### 4.3 Related terms

Related terms share conceptual similarities with the focal concept of IPCP but lack its defining attributes (Rodgers and Knafl, 2000; Tofthagen and Fagerstrøm, 2010). Examples include interprofessional education (IPE), interprofessional patient-centered (IPPC) practice, interprofessional collaboration, and interdisciplinary collaboration. These distinctions are further examined in the literature reviewed for this analysis. In 2015, the Institute of Medicine highlighted promising evidence regarding the impact of IPE on patient, population, and health system outcomes (Brashers et al., 2015). Although IPE supports skill development, its direct linkage to patient outcomes remains difficult to establish due to challenges in measuring collaborative practice (Brashers et al., 2015; Cox et al., 2016; Zwarenstein et al., 2009).

Interprofessional patient-centered (IPPC) practice emphasizes a culture of caring that positions patients as active team members that prioritizes patient involvement as an integral member of the team (Bilodeau et al., 2015; Dahlke et al., 2020; Reeves et al., 2017; Wei et al., 2020). While aligned conceptually, IPCP more explicitly integrates this engagement into enacted collaborative care.

Given these limitations, existing definitions do not fully capture IPCP as a distinct, operational construct. Therefore, an operational definition is proposed that conceptualizes IPCP as an observable form of collaborative team functioning with potential relevance for future implementation, evaluation, and measurement development. IPCP is defined as collaborative team functioning enacted through shared decision-making, reciprocal relationships, role clarity, and coordinated communication among healthcare professionals, patients, and families to achieve safe, effective, and person-centered care. Following Rodgers and Knafl (2000) method of evolutionary concept analysis, antecedents, attributes, and consequences identified in relation to IPCP are defined accordingly.

## 5 Results

The following sections present the antecedents, attributes, consequences, and exemplars identified through this concept analysis. Consistent with Rodgers’evolutionary method, these components reflect patterns most consistently represented across the 43 sources reviewed, synthesized qualitatively rather than through the formal frequency counts characteristic of systematic reviews. Table 2 summarizes the frequency with which each antecedent, attribute, and consequence of IPCP was identified across the 43 included sources.

**TABLE 2:** Frequency of antecedents, attributes, and consequences of interprofessional collaborative practice across included sources (*N* = 43).

Category	Component	n	Supporting sources (Table 1 article number)
Terminology	Definitions	7	1, 4, 11, 17, 25, 29, 30
	Surrogate terms	4	1, 3, 4, 27
	Related concepts	15	1, 2, 4, 6, 9, 10, 11, 14, 16, 17, 22, 25, 27, 29, 39
Antecedent	Collaborative practice-ready workforce	27	3, 4, 6, 8, 9, 11, 14, 15, 16, 17, 19, 20, 23, 26, 28, 30, 35, 37, 38, 42
	Clear roles and responsibilities	28	4, 5, 7, 8, 10, 11, 12, 14, 15, 18, 19, 21, 23, 24, 26, 28, 29, 30, 31, 33, 35, 36, 38, 39, 40
	Communication	39	2, 3, 4, 5, 7, 8, 10, 12, 13, 14, 15, 18, 19, 21, 22, 24, 25, 26, 27, 28, 29, 30, 31, 32, 33, 35, 36, 37, 38, 39, 40, 43
	Developing trust	19	4, 8, 14, 17, 18, 19, 21, 26, 33, 37, 38, 39, 40, 41, 42, 43
	Shared mental model	17	4, 5, 6, 11, 15, 17, 18, 19, 22, 25, 31, 38, 40, 42, 43
Attribute	Shared decision-making	28	2, 4, 7, 8, 12, 13, 14, 15, 17, 18, 19, 21, 25, 26, 28, 30, 31, 33, 35, 36, 38, 39, 40
	Individualized care planning	13	2, 4, 7, 10, 12, 14, 18, 26, 30, 35, 36, 38, 43
	Promoting mutual respect	24	2, 5, 8, 10, 11, 14, 17, 19, 20, 21, 26, 30, 31, 35, 37, 38, 39, 40, 41, 42, 43
	Promoting reciprocal relationships	9	2, 8, 37, 38, 39, 41, 43
Consequence	Enhanced role awareness	9	2, 11, 18, 23, 26, 35, 36, 37, 41
	Team empowerment	17	4, 8, 12, 14, 19, 21, 25, 26, 30, 31, 38, 39, 40, 42, 43
	Improved care outcomes	29	6, 7, 8, 9, 12, 13, 14, 15, 16, 17, 19, 20, 21, 22, 25, 26, 27, 29, 30, 31, 33, 34, 35, 38, 39, 40, 43
	Mutuality among all team members	25	4, 7, 9, 10, 12, 13, 14, 17, 18, 19, 26, 28, 30, 32, 35, 36, 38, 39, 40, 41, 43

Operational definitions for each antecedent, attribute, and consequence, and the criteria used for source articles are provided in Supplementary Table 1: Literature Matrix Codebook.

### 5.1 Conceptual model

The synthesis of IPCP elements is represented in the IPCP conceptual model (Figure 2), illustrating the dynamic relationships among antecedents, attributes, consequences, and exemplars. The model reflects IPCP as a fluid, evolving construct, consistent with Rodgers’ evolutionary approach. The sunrise metaphor represents ongoing emergence and development, emphasizing IPCP as a continuously evolving practice shaping collaborative care.

**FIGURE 2:**
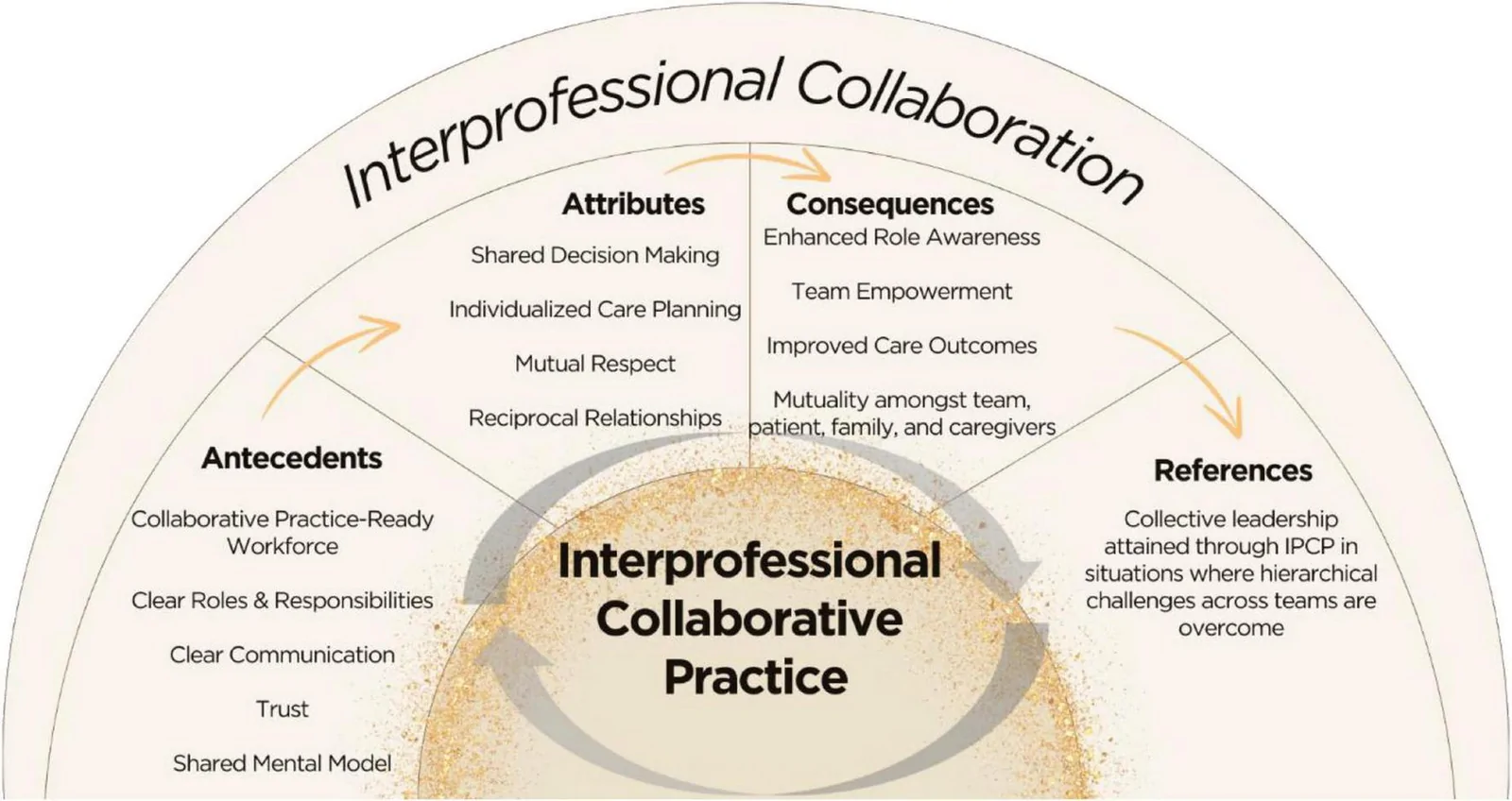
Conceptual model of interprofessional collaborative practice.

The model supports contextual adaptability across healthcare systems while maintaining conceptual coherence. While most of the findings stem from North American and European publications, the attributes, antecedents, and consequences may be differently expressed across international contexts. Accordingly, the model supports adaptability while maintaining conceptual coherence, reflecting both specificity and flexibility within evolving healthcare systems.

Viewed through a Caring Science lens, IPCP is grounded in relational practice and a culture of caring (Wei et al., 2020). Through the lens of evidence-based nursing theory (Chinn and Kramer, 2014), the application of IPCP has the potential to enhance patient care outcomes (Cox et al., 2016; Girard, 2021; Reeves et al., 2017). A collective ownership mentality fosters a culture of caring within interprofessional practice teams (Bilodeau et al., 2015; Wei et al., 2020; Yamamoto et al., 2022). IPCP aligns with Convergent Care Theory (Wei, 2021), encompassing (1) all-inclusive organizational care, (2) healthcare professional collaborative care, (3) person-centered precision care, and (4) patients’ and healthcare providers’ self-care. Through competence, compassion, accountability, trusting, sharing, and engaging, convergent care is achieved across interprofessional teams (Wei, 2021).

### 5.2 Antecedents

Antecedents of the concept of IPCP occur prior to realization of the concept. Results reveal antecedents for IPCP include: (1) a collaborative practice-ready workforce, (2) clear roles and responsibilities, (3) communication, (4) developing trust, and (5) a shared mental model.

#### 5.2.1 Collaborative practice-ready workforce

As healthcare continues to evolve toward a team-based, person-centric model, there is growing emphasis on collaborative ways of knowing to support a coordinated team approach to care (Venville and Andrews, 2020). A workforce prepared for collaboration is the foundation of IPCP. Interprofessional education (IPE) plays a foundational role in equipping health professionals with the knowledge, skills, and attitudes necessary to meet the diverse and complex healthcare needs of their communities (Corbaz-Kurth et al., 2026; Green and Johnson, 2015; World Health Organization, 2010). Collaborative behavior targeted as a learning outcome by 70.7% of participating teams (Blakeney et al., 2021).

Three interrelated components contribute to development of this collaborative practice-ready workforce (Dolan and Nowell, 2025; Reinders and Krijnen, 2023; Venville and Andrews, 2020): interprofessional education, which builds foundational collaborative knowledge and skills (Brashers et al., 2015; Cox et al., 2016; Gautama et al., 2023; McGreal et al., 2023); enculturation, the socialization process through which these skills and values are internalized within a discipline and across a team (Rodgers and Knafl, 2000); and interprofessional identity, an individual’s internalized sense of self as a collaborative team member (Reinders and Krijnen, 2023; Ward et al., 2017; Wood et al., 2025). Interprofessional education (IPE), professional enculturation, and the development of an interprofessional identity function as complementary preparatory processes that collectively cultivate the knowledge, skills, values, behaviors, and professional orientation required for effective interprofessional collaborative practice. These processes collectively contribute to the development of a workforce that is prepared to engage in collaborative, person-centered care across healthcare settings.

##### 5.2.1.1 Interprofessional education

Pre-clinical interprofessional education (IPE) initiatives at the undergraduate level help students develop teamwork skills, understand diverse healthcare roles, and acquire essential knowledge for delivering person-centric care (Brashers et al., 2015; Corbaz-Kurth et al., 2026; Cox et al., 2016; Dolan and Nowell, 2025; Gautama et al., 2023; Interprofessional Education Collaborative, 2023; MacMillan and Reeves, 2014; McGreal et al., 2023; Wood et al., 2025). In the author’s interpretation of article review, pre-clinical preparation ensures a smooth transition of new graduate professionals into the clinical workplace, thereby reducing the cognitive load associated with integrating into a complex and diverse healthcare team (Brashers et al., 2015; Cox et al., 2016; Gautama et al., 2023; Interprofessional Education Collaborative, 2023; MacMillan and Reeves, 2014; McGreal et al., 2023; Wood et al., 2025). IPE is directly identified as an antecedent to interprofessional collaboration in a earlier concept analysis (Bookey-Bassett et al., 2017), highlighting the significant link between IPE and IPCP. Through IPE, interprofessional readiness and interprofessional socialization prepare nurses and other health professionals to function as a collaborative practice-ready workforce (Dolan and Nowell, 2025; McGreal et al., 2023; Venville and Andrews, 2020).

##### 5.2.1.2 Enculturation

Rodgers describes the integration of a concept within a discipline as a process of enculturation: the internalization of shared meanings through socialization (Rodgers and Knafl, 2000). In parallel, interprofessional enculturation or interprofessional socialization (Dolan and Nowell, 2025) is essential for integrating healthcare professionals into IPCP, fostering safe, efficient, and person-centric care (Dolan and Nowell, 2025; Hall, 2005; McGreal et al., 2023). This is an ongoing process in which individuals from diverse professional backgrounds engage collaboratively, recognizing and valuing each other’s unique contributions to the team (Dolan and Nowell, 2025). In the author’s interpretation, interprofessional enculturation involves actively dismantling misconceptions and professional biases, while cultivating a dual identity: one that honors both individual professional roles and a collective interprofessional identity in support of a collaborative, practice-ready workforce (Dolan and Nowell, 2025; Reinders and Krijnen, 2023).

##### 5.2.1.3 Interprofessional identity

Understanding one’s interprofessional identity is increasingly recognized in the literature as a foundational element in developing a collaborative practice-ready workforce (Reinders and Krijnen, 2023; Ward et al., 2017; Wood et al., 2025). Cultivating this identity within clinical teams enhances future collaborative performance and supports individuals in confidently participating as integral members of healthcare teams (Reinders and Krijnen, 2023). The understanding of one’s self is a precursor to understanding one’s interprofessional self prior to embarking on complex interprofessional encounters (Ward et al., 2017). Strong professional identity and role clarity enable interprofessional team members to engage meaningfully in shared decision-making, strengthening professional competence and integrated care planning (O’Donnell et al., 2025). Conversely, the literature associates an unclear interprofessional identity with reduced role understanding and diminished capacity to contribute meaningfully to team-based care (Reinders and Krijnen, 2023; Wood et al., 2025). As interpreted from the literature, without a clear sense of interprofessional identity, understanding one’s role, responsibilities, and capacity to contribute meaningfully to a team approach, care becomes increasingly difficult.

#### 5.2.2 Clear roles and responsibilities

Clearly defined roles and responsibilities are essential for safe and effective care (American Nurses Association, 2025; Cao et al., 2018; Dahlke et al., 2020; Interprofessional Education Collaborative, 2023; Matziou et al., 2014; Milton et al., 2022; Verhaegh et al., 2017). Sustained discussion of role overlap and boundary tension is a persistent, unresolved problem across interprofessional teams (Fox and Reeves, 2015; Schot et al., 2020). The Interprofessional Education Collaborative (2023) identifies Roles and Responsibilities as one of the Core Competencies for Interprofessional Collaborative Practice, emphasizing the importance of understanding both one’s own role and the expertise of team members to optimize patient and population health outcomes (Interprofessional Education Collaborative, 2023).

The American Heart Association (AHA; American Heart Association, 2020) highlights clear role assignment as one of the eight elements of effective team dynamics in high-performance resuscitation teams. Clearly assigning responsibilities ensures that tasks are performed promptly and skillfully by the most qualified team member, enhancing both efficiency and outcomes during critical events such as resuscitations (American Heart Association, 2020). When team members clearly understand their own roles and those of others, and are prepared to fulfill their responsibilities, collaboration is optimized (Cao et al., 2018). Conversely, role ambiguity contributes to duplication, missed tasks, and reduced team performance (American Heart Association, 2020; American Nurses Association, 2025). In contrast, role clarity enables coordinated action, strengthens teamwork, and improves clinical outcomes (American Heart Association, 2020; Dahlke et al., 2020; Gilfoyle et al., 2017; Interprofessional Education Collaborative, 2023; Schuetz Haemmerli et al., 2021; World Health Organization, 2010).

#### 5.2.3 Communication

Effective communication is consistently identified as foundational to successful interprofessional collaborative practice (American Heart Association, 2020; Bilodeau et al., 2015; Bookey-Bassett et al., 2017; Dahlke et al., 2020; Gausvik et al., 2015; Gilfoyle et al., 2017; Interprofessional Education Collaborative, 2023; Schwartz et al., 2024; Sillero Sillero and Buil, 2021; Stewart, 2018; Verhaegh et al., 2017; World Health Organization, 2010; Yamamoto et al., 2022; Zwarenstein et al., 2009). Good, effective, and frequent team communication is identified as a defining characteristic of effective IPCP teams (Blakeney et al., 2021; Bookey-Bassett et al., 2017; Reeves et al., 2017; Schuetz Haemmerli et al., 2021). Breakdowns in communication are strongly associated with adverse patient outcomes (Schwartz et al., 2024; Shirey et al., 2021; Stewart, 2018). Schot et al. (2020) identified bridging communicational divides as a core professional contribution to interprofessional teams, encompassing the active transfer of knowledge and information between professionals, translation of discipline-specific language into terms other team members can use, and deliberate efforts to remain available to colleagues, particularly across geographically fragmented care settings (Schot et al., 2020). In complex care environments, diverse interprofessional teams are particularly vulnerable to miscommunication due to competing work demands, distractions, and time constraints (Sillero Sillero and Buil, 2021).

Matziou et al. (2014) emphasize the pivotal role of interprofessional communication in promoting high-quality patient care and underscore the importance of nurses’ active participation in clinical decision-making. However, physicians often cite the lack of recognition of nurses’ professional roles as a barrier to effective collaboration (Matziou et al., 2014). When interprofessional communication is poor, the likelihood of errors and omissions in care increases, posing significant risks to patient safety (Matziou et al., 2014). Nurses face unique challenges in managing communication within teams due to both physical and psychosocial demands of clinical settings, yet it remains essential that these challenges do not compromise care delivery (Bilodeau et al., 2015; Matziou et al., 2014; Milton et al., 2022). Clear and effective communication between team members is identified as an essential element of providing patient-centered care (Dahlke et al., 2020).

Clear, respectful, and composed communication is critical to sustaining high-performance team interactions, particularly in high-acuity clinical situations. Interprofessional miscommunication is identified as a central driver of poor patient outcomes, describing how patients caught between conflicting professional opinions can become confused about whose clinical advice to follow and, ultimately, whom to trust (Stewart, 2018). Constructive intervention is the proactive act of addressing potential issues in patient care by speaking up before an error occurs, regardless of one’s role in the team (American Heart Association, 2020). It is a crucial safeguard, ensuring accountability and preventing mistakes through open communication and collaborative problem-solving. This is a crucial component of patient safety, emphasizing a culture of speaking up to prevent errors, whether as a team member or team leader (American Heart Association, 2020). Clear messages, closed-loop communication, and constructive intervention also serve as strategies for effective team dynamics (American Heart Association, 2020). These relational strategies reflect a way of being grounded in relational presence, mutual respect, and authentic engagement among team members (Cara, 1999; Watson, 2008). Within this framework, open communication is described as a means for healthcare professionals to move beyond their own clinical perspectives and engage with the patient’s unique worldview, supporting shared meaning-making as a team (Dolan and Nowell, 2025; Wood et al., 2025).

#### 5.2.4 Developing trust

Trusting-caring relationships foster a climate grounded in respect for human dignity, relational integrity, and authentic connectedness (Corbaz-Kurth et al., 2026; Green and Johnson, 2015; Watson, 2008; Wei and Watson, 2019; Wei et al., 2020). Trust is a critical antecedent to interprofessional collaborative practice, enabling the psychological safety and relational integrity necessary for collaboration across professional boundaries (Bookey-Bassett et al., 2017; Corbaz-Kurth et al., 2026; Gausvik et al., 2015; Matziou et al., 2014). Establishing a trusting environment requires clear communication, psychological safety (PS), and shared decision-making (American Heart Association, 2020; Corbaz-Kurth et al., 2026; Matziou et al., 2014; Wei and Watson, 2019; Wei et al., 2020; Wei, 2021). PS is defined as a subjective perception that the environment is safe for interpersonal risk-taking, enabling individuals to speak up, ask questions, or acknowledge mistakes without fear of embarrassment or judgment (Edmondson and Bransby, 2023; Edmondson, 1999; Edmondson and Lei, 2014). Identify high-quality, regular, and frequent communication as the most significant precondition for trust across teams, where trust is explicitly described as a reciprocal and contagious phenomenon while self-confidence and trust reinforce each other bidirectionally between colleagues (Corbaz-Kurth et al., 2026).

Trust fosters mutual respect and reciprocal relationships across interprofessional teams. It is foundational both to effective collaboration and patient-provider relationships, where it enhances patient engagement and outcomes. Viewed through a caring-healing lens, IPCP emerges as a dynamic, cyclical process in which trust is continuously reinforced through authentic, non-hierarchical human connection (Hall, 2005; Ward et al., 2017). Trust propagates convergent care across teams, strengthening collaboration, enhancing communication, and improving team effectiveness and clinical outcomes (American Heart Association, 2020; Dahlke et al., 2020; Gausvik et al., 2015; Interprofessional Education Collaborative, 2023; Schuetz Haemmerli et al., 2021; Wei and Watson, 2019; Wei, 2021). Sustained trust therefore underpins the development of high-performing, compassionate, person-centric teams within a culture of caring.

#### 5.2.5 Shared mental model

Shared mental models refer to the collective understanding and mutual cognitive framework within a team, aiding members in collaborative functioning within their respective contexts (Brashers et al., 2015; Brashers et al., 2020; Dolan and Nowell, 2025; Green and Johnson, 2015; McComb and Simpson, 2014; Wood et al., 2025). A collective is developed over time as interprofessional teams continue to build interpersonal relationships (Yamamoto et al., 2022). A shared mental model allows interprofessional collaborative teams to align their understanding, expectations, and goals, thereby facilitating coordinated person-centric care. Sillero Sillero and Buil (2021) similarly identified team mental model sharing as a strategy for overcoming interprofessional collaboration barriers, describing structured operating room briefings as an opportunity for surgical team members to share mental models and improve patient safety (Sillero Sillero and Buil, 2021). In IPE, the development of a shared mental model is foundational to team integration and relational effectiveness (Brashers et al., 2015, 2020). Interprofessional socialization fosters a shared understanding by aligning professionals around common values and goals (Dolan and Nowell, 2025). Shared mental models enhance interprofessional collaboration by fostering understanding, coordinated action, and improved team performance (Gilfoyle et al., 2017; McComb and Simpson, 2014; Wood et al., 2025). This promotes successful teamwork and collaboration across healthcare teams, enhancing the quality and safety of patient care.

### 5.3 Attributes

Defining attributes are the core characteristics that distinguish IPCP from related or surrogate terms or forms of interprofessional collaboration (Rodgers and Knafl, 2000). Attributes encompass terms that the literature describes a concept in actual practice (Tofthagen and Fagerstrøm, 2010). Findings of this analysis revealed attributes of IPCP to include: (1) shared decision making, (2) individualized care planning, (3) mutual respect, and (4) reciprocal relationships.

#### 5.3.1 Shared decision-making

Shared decision-making within non-hierarchical teams promotes empowerment and effective IPCP (Bilodeau et al., 2015; Bookey-Bassett et al., 2017; Fox and Reeves, 2015; Matziou et al., 2014; Sillero Sillero and Buil, 2021; Verhaegh et al., 2017). When hierarchies are minimized, patients are positioned as active partners in care, contributing to collaborative decision-making (Green and Johnson, 2015; World Health Organization, 2010). Trust supports shared decision-making by enabling professionals to engage openly, acknowledge vulnerability, and rely on one another’s competence in complex clinical situations (Corbaz-Kurth et al., 2026). Within high-performance teams, the team leader plays a crucial role in fostering shared leadership, ensuring members actively engage rather than passively follow directions. The practice of knowledge sharing is a critical component of effective team performance especially when a team leader is involved in fixation error (American Heart Association, 2020). By being focused on a specific treatment or diagnostic approach, essential information is overlooked (American Heart Association, 2020). While the physician is often considered the default team leader, effective IPCP emphasizes the patient’s role as a central contributor to their care. In a person-centric model, patients are encouraged to participate in decisions about their treatment, shifting from passive recipients to active co-leaders in the care process (Bilodeau et al., 2015). These defining attributes are reinforced by a realist synthesis (O’Donnell et al., 2025) and empirical studies (Schuetz Haemmerli et al., 2021; Reinders and Krijnen, 2023) demonstrating that shared decision-making, meaningful inclusion of patients and caregivers, and team learning cultures function as key mechanisms through which interprofessional collaboration is enacted in practice (O’Donnell et al., 2025; Reinders and Krijnen, 2023; Schuetz Haemmerli et al., 2021).

Sharing leadership strengthens interprofessional bonds and promotes shared accountability among team members (Bilodeau et al., 2015; Schuetz Haemmerli et al., 2021; Venville and Andrews, 2020; Wei et al., 2020). Sharing clinicians adopt two-way communication, inviting patients to contribute insights and preferences into clinical dialogue (Stewart, 2018). Perspectives on patient involvement in decision-making varies across professional roles, highlighting the need for continued dialogue and role clarification (Bilodeau et al., 2015; Verhaegh et al., 2017). This is further supported by the recommendation that the inclusion of patient, family, and caregiver experiences places patients and families as valued contributors (Cox et al., 2016).

Structured Interdisciplinary Bedside Rounds (SIBR) exemplifies this attribute, offering a practical, evidence-based model that operationalizes IPCP in practice. SIBRs improve interprofessional communication, workflow efficiency, and provider and patient/family satisfaction (Cao et al., 2018; Gausvik et al., 2015; Schwartz et al., 2024). SIBR are standardized, interprofessional team-based interventions for delivery of high-quality patient care (Schwartz et al., 2024). They facilitate real-time collaboration and transparency, enhancing team cohesion and the quality of care delivered. While SIBR have demonstrated improved perceptions of IPCP, their specific, measurable impact on IPCP remains underexplored. Nevertheless, SIBR have been associated with improvements in communication quality, patient safety, nursing satisfaction, and nurse retention, making this a valuable model for embedding IPCP in complex clinical environments (Cao et al., 2018; Gausvik et al., 2015; Schwartz et al., 2024). By offering a structured, person-centered approach to teamwork, SIBRs provide a tangible approach to implementing IPCP across dynamic healthcare teams.

#### 5.3.2 Individualized care planning

Individualized care planning integrates professional expertise with patient preferences. Despite this ideal, tensions often arise between professional-centered models and the goals of person-centered, individualized care, highlighting the need for more equitable team dynamics and shared decision-making (Bookey-Bassett et al., 2017; Fox and Reeves, 2015). A persistent paradox exists between the ideals of person-centered discourse and the reality of professional-centered practices (Bilodeau et al., 2015). Hierarchical dynamics impede the realization of individualized care goals both among health professionals and between professionals and patients (Fox and Reeves, 2015). Person-centered precision care is supported by shared decision-making across interprofessional teams and facilitates individualized care planning (Dahlke et al., 2020; Fox and Reeves, 2015; Wei, 2021). Emerging models of interprofessional practice support tailored approaches, integrating diverse professional expertise to co-create personalized care strategies alongside the patient and family (Gautama et al., 2023; Schuetz Haemmerli et al., 2021). IPCP enables co-creation of care plans aligned with patient goals, strengthening both autonomy and collaboration.

Authentic human connection within interprofessional patient-centered (IPPC) practice is essential to delivering truly individualized care. This approach places the patient at the center of their care, giving them power to act as the leader of their own healthcare team (Bilodeau et al., 2015). The patient draws upon the expertise of healthcare professionals through reciprocal knowledge-sharing, contributing to informed, collaborative decision-making. This model of individualized care planning supports patient autonomy and strengthens interprofessional collaboration (Fox and Reeves, 2015; Bilodeau et al., 2015; Dahlke et al., 2020), enabling teams to co-create care strategies that are person-centric and clinically sound. By articulating individual needs, patients guide the care team in delivering care that is aligned with their goals and values.

#### 5.3.3 Promoting mutual respect

Mutual respect underpins effective interprofessional collaboration for optimal team performance (American Heart Association, 2020; American Nurses Association, 2025; Brashers et al., 2020; Dahlke et al., 2020; Gausvik et al., 2015; Green and Johnson, 2015; Interprofessional Education Collaborative, 2023). When team members value each other’s roles, expertise, and contributions, they create a culture in which optimal team performance becomes possible. The IPEC Core Competencies for Interprofessional Collaborative Practice highlight the Values of Ethics in working with one another to maintain a climate of shared values, ethical conduct, and mutual respect (Brashers et al., 2020; Interprofessional Education Collaborative, 2023). Ethical practice, grounded in shared values and mutual accountability, extends beyond patient-provider interactions to include how healthcare professionals engage with one another. Promoting empathy, compassion, psychological safety, and confidentiality within teams contributes to a just and caring culture that protects both the dignity of patients and the well-becoming of providers. When these principles are intentionally embedded into everyday practices of healthcare teams, they cultivate trust through a sustainable, high-functioning workforce within a just culture of caring (Bilodeau et al., 2015; Interprofessional Education Collaborative, 2023; Wei and Watson, 2019; Wei et al., 2020). Interprofessional teams therefore cultivate trust, enhance communication, and improve collaborative decision-making, leading to better patient outcomes and a more resilient healthcare workforce (Bilodeau et al., 2015; Interprofessional Education Collaborative, 2023; Wei et al., 2020; Wei and Watson, 2019).

High-performance teams thrive when built upon a foundation of mutual respect, collegiality and shared accountability, fostering a supportive interprofessional environment (American Heart Association, 2020; Brashers et al., 2020; Interprofessional Education Collaborative, 2023). Effective collaboration extends beyond professional expertise and requires a shift in mindset where hierarchical barriers are dismantled, and team members engage as equal partners in care. A lack of mutual respect and authentic collaboration has been linked to disjointed care coordination (MacMillan and Reeves, 2014). For interprofessional teams to function optimally, individual egos and professional pride must not overshadow collective goals or hinder effective teamwork (Wei et al., 2020). Professional humility and shared leadership are similarly associated with strengthened team dynamics, communication, and decision-making (Schuetz Haemmerli et al., 2021; Wei et al., 2020).

#### 5.3.4 Promoting reciprocal relationships

Establishing reciprocal caring relationships across diverse healthcare teams is a key catalyst for fostering positive IPCP experiences (Bilodeau et al., 2015; Corbaz-Kurth et al., 2026; Ward et al., 2017; Wei and Watson, 2019; Wei et al., 2020; Yamamoto et al., 2022). By recognizing and leveraging each team member’s unique strengths, healthcare teams develop deeper trust, stronger cohesion, and a culture of continuous learning and mutual support. This includes that of patients, professionals, and the overall team, promoting two-way reciprocity (Bilodeau et al., 2015). This reciprocity contributes to improved team performance, increased job satisfaction, and higher retention rates, particularly significant in high-stress clinical environments where burnout is prevalent (Moss et al., 2016; Welp et al., 2016; West et al., 2018).

### 5.4 Consequences

Consequences encompass the positive or negative outcomes of IPCP, and the result of the use of a IPCP in a practical situation (Rodgers, 2000; Tofthagen and Fagerstrøm, 2010). This analysis identifies the consequences of IPCP as: (1) enhanced role awareness, (2) team empowerment, (3) improved care outcomes, and (4) mutuality among the team, patients, and families. The team dynamic of interprofessional collaborative care delivery changes from context to context, with diverse teams coming together to care for diverse patients in need of complex care.

#### 5.4.1 Enhanced role awareness

Greater understanding of roles strengthens confidence, coordination, and collaboration, improving team effectiveness. Role awareness, achieved through understanding one’s own responsibilities and fostering mutual professional relationships, enhances a team’s capacity for effective interprofessional collaboration (American Heart Association, 2020; McGreal et al., 2023; World Health Organization, 2010). Enhanced role awareness is associated with improved confidence and competence within the team, eliminating misunderstandings and improving awareness of others’ roles (McGreal et al., 2023). Empowering healthcare professionals similarly empowers patients and families by clarifying their role in care through shared decision-making, individualized planning, mutual respect, and reciprocal relationships (Schuetz Haemmerli et al., 2021; Verhaegh et al., 2017; Wei and Watson, 2019).

#### 5.4.2 Team empowerment

In this analysis, team empowerment emerges from shared leadership and inclusive decision-making in which empowered teams demonstrate stronger collaboration, resilience, and engagement, contributing to improved care quality. Team empowerment is foundational to high-functioning interprofessional teams, reflecting the extent to which team members experience shared decision-making, mutual accountability, trust, and inclusive leadership across disciplines (Corbaz-Kurth et al., 2026; Sillero Sillero and Buil, 2021). Engaged teams are empowered and are more likely to co-create care planning, integrate diverse professional perspectives, and promote both patient and provider agency (Fox and Reeves, 2015; Bilodeau et al., 2015; Dahlke et al., 2020). Nurses in particular express a strong desire to be more involved in clinical decisions, underscoring the importance of organizational and structural recognition and support of their contributions within interprofessional teams (Matziou et al., 2014).

Team empowerment is strengthened through informal communication, shared understanding, and trust as demonstrated in primary care settings where collaborative relationships enhance collective decision-making (Morgan et al., 2015). Environments that support professional growth further reinforce team engagement, empowerment and interprofessional effectiveness (Gautama et al., 2023). Empowered interprofessional teams are reported to demonstrate greater collaboration and care quality, alongside more resilient, inclusive team cultures (Gautama et al., 2023; Morgan et al., 2015).

Through development of an ownership mentality, empowered teams are more likely to enact shared leadership, regardless of title, role or profession (Wei et al., 2020). Interprofessional, trauma-informed approaches support patients’ psychological, physical, and emotional safety while promoting choice, control, and empowerment through collaborative, person-centered care (Lapum et al., 2020). Team empowerment arising from shared leadership and advocacy is associated with higher satisfaction among staff, patients, and their families (Dahlke et al., 2020).

#### 5.4.3 Improved care outcomes

The incorporation of IPCP is associated with improved care outcomes when implemented effectively across teams (American Nurses Association, 2025; Brashers et al., 2015; Corbaz-Kurth et al., 2026; Cox et al., 2016; Gilfoyle et al., 2017; Green and Johnson, 2015; Interprofessional Education Collaborative, 2023; Reeves et al., 2017; Schot et al., 2020; Shirey et al., 2021; Utley et al., 2020; Wei et al., 2022; World Health Organization, 2010; Zwarenstein et al., 2009), though this concept analysis does not, on its own, establish a causal relationship. For example, an interprofessional collaborative practice model for transitional care coordination in underserved populations with diabetes and heart failure demonstrated measurable improvements in clinical indicators, sustained patient satisfaction and substantial reductions in emergency department visits, hospital readmissions, and length of stay, translating into significant cost avoidance for the health system (Shirey et al., 2021). Teams proficient in collaborative practice work synergistically to improve patient care, reduce errors, enhance safety, and elevate overall care quality and outcomes (Brashers et al., 2015; Corbaz-Kurth et al., 2026; Green and Johnson, 2015; Interprofessional Education Collaborative, 2023; World Health Organization, 2010). Benefits extend beyond patients to include staff satisfaction and reduced burnout (Gausvik et al., 2015).

#### 5.4.4 Mutuality among all team members

Mutuality is fostered through shared decision-making and individualized care planning grounded in mutual respect and reciprocal relationships. Mutuality is further highlighted through involving family members in the interprofessional team (Bookey-Bassett et al., 2017; Cao et al., 2018; Fox and Reeves, 2015; Venville and Andrews, 2020). Through Structured Interdisciplinary Bedside Rounds (SIBR), shared leadership enables individualized decision-making, with patients positioned as active leaders of their care (Cao et al., 2018; Gausvik et al., 2015; Schwartz et al., 2024). This approach to navigating patient and family involvement in care generates mutual benefit across teams, enhancing workplace satisfaction, strengthening team functioning, and improving patient outcomes (Dahlke et al., 2020).

The development of a collective leadership attained through optimal IPCP is essential specifically in contexts, cultures, or situations where hierarchical challenges across teams are encountered (Schuetz Haemmerli et al., 2021; Sillero Sillero and Buil, 2021; Yamamoto et al., 2022). Nevertheless, stratifications and hierarchical distinctions remain among health professions (Reinders and Krijnen, 2023; Lingard et al., 2004). A flattened organizational approach to interprofessional collaborative practice is described in the literature as supporting equity across the team, positioning all members, including patients and families, as valued contributors (Baggs, 2022; Cao et al., 2018; Ward et al., 2017; Wei, 2021; Yamamoto et al., 2022).

### 5.5 Operationalizing interprofessional collaborative practice

The findings of this concept analysis suggest that IPCP may be operationalized through observable collaborative behaviors and enacted team processes. Potential indicators include role clarity, shared decision-making, interprofessional communication, patient and family engagement, mutual respect, reciprocal relationship-building, trust, and collaborative leadership behaviors (Table 3). Collectively, these indicators provide a foundation for future development of measurement instruments and evaluation frameworks capable of assessing IPCP as a distinct construct within healthcare teams. By translating conceptual components into operational and observable practices, IPCP may be examined, implemented, and evaluated across diverse healthcare settings.

**TABLE 3:** Proposed operational indicators of interprofessional collaborative practice.

IPCP component	Proposed operational indicator
Role clarity	Understanding of team roles and responsibilities
Shared decision-making	Participation in collaborative treatment planning
Interprofessional communication	Closed-loop communication behaviors
Patient and family engagement	Patient and family participation in rounds and care planning
Trust	Psychological safety and mutual reliance among team members
Collaborative leadership	Shared leadership and collective decision-making behaviors

### 5.6 References and exemplars of IPCP

References are the context or situations in which a concept occurs (Rodgers and Knafl, 2000). IPCP frequently appears in the literature within clinical environments, and is not limited to a specific context or patient population (Abu-Rish Blakeney et al., 2019; Bilodeau et al., 2015; Blakeney et al., 2021; Bookey-Bassett et al., 2017; Cao et al., 2018; Gausvik et al., 2015; Gilfoyle et al., 2017; Schwartz et al., 2024; Stewart, 2018). Collective leadership attained through well-established IPCP occurs in situations where hierarchical challenges across teams are overcome. Critical care settings provide prominent references where collaboration is essential to manage complexity (Lingard et al., 2004). A caring culture further serves as a protective mechanism that helps teams function well in complex contexts, consistent with the theoretical nature outlined above. Even amid the obstacles of critical situations, person-centric interprofessional care is described in the literature as supporting teams’capacity to work through challenges by establishing a shared sense of collective identity (Wei, 2021; Wei and Watson, 2019).

An exemplar serves to provide a practical example of the concept within the context of interest (Rodgers and Knafl, 2000; Tofthagen and Fagerstrøm, 2010). The exemplar does not serve to provide a model case of the concept of choice, yet provides examples of the concept extracted from the data resulting from the integrative literature search conducted (Rodgers and Knafl, 2000; Tofthagen and Fagerstrøm, 2010). An exemplar of IPCP is demonstrated in Figure 3, demonstrating implementation within a high-performing interprofessional team. Structured Interdisciplinary Bedside Rounds (SIBR) emerged as a key exemplar of interprofessional collaborative practice. Although described as interdisciplinary in the original literature, SIBR demonstrates the defining attributes of IPCP through shared decision-making, collaborative leadership, role clarity, interprofessional communication, and patient engagement. The incorporation of SIBR reinforces the antecedents, attributes, and consequences of practical implementation of IPCP in a clinical setting. Structured Interdisciplinary Bedside Rounds operationalize IPCP by integrating communication, shared decision-making, and patient engagement.

**FIGURE 3:**
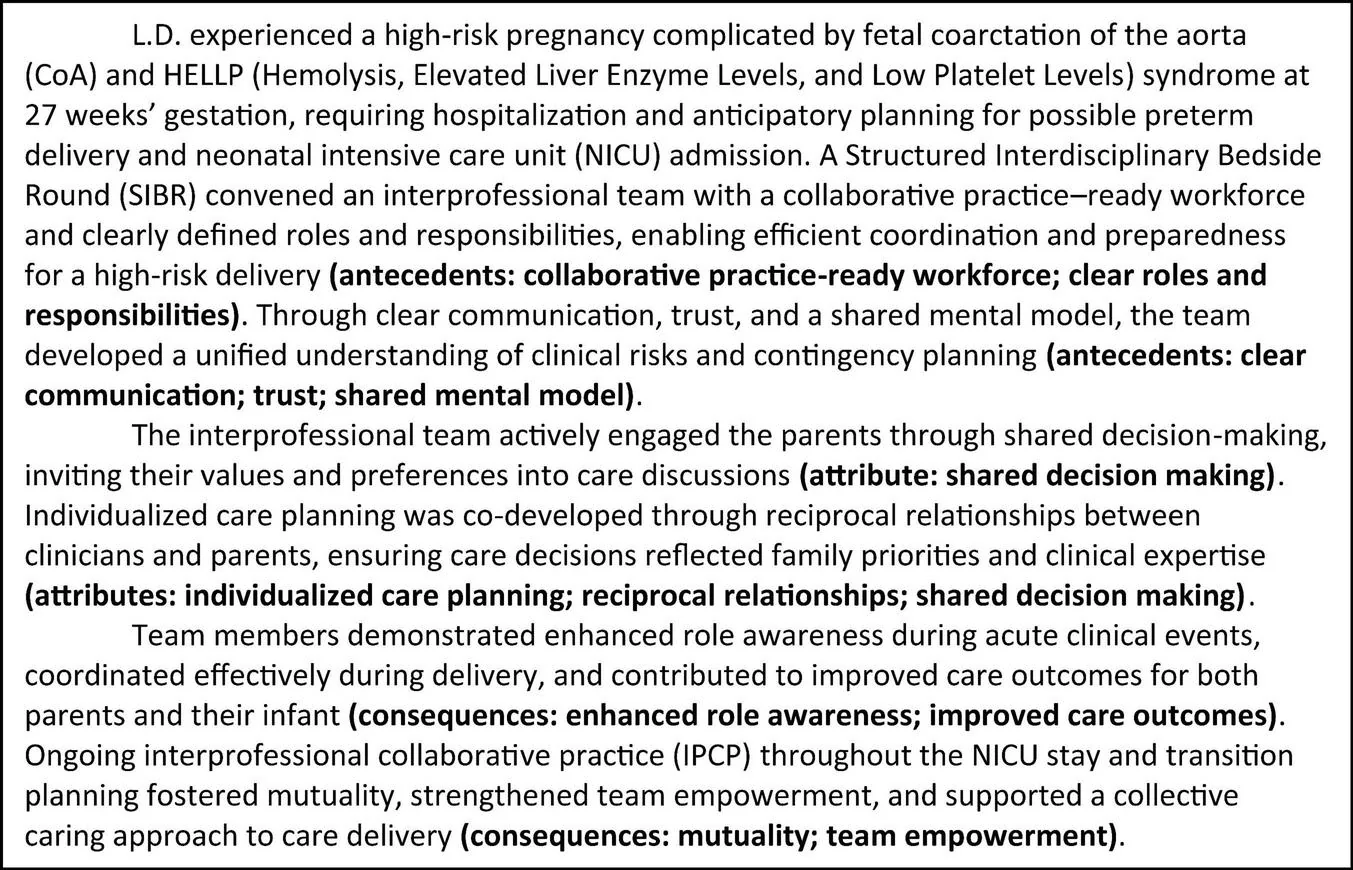
Structured interdisciplinary bedside rounds as an exemplar of operationalized interprofessional collaborative practice in critical care.

## 6 Discussion

### 6.1 Implications

This concept analysis extends beyond conceptual clarification to provide a foundation for future work operationalizing interprofessional collaborative practice across research, practice, policy, and education. A clearer, more operational definition may support more consistent implementation and evaluation of collaborative, team-based, person-centered care. However, this analysis identifies candidate antecedents, attributes, consequences, and indicators rather than establishing causal or measured relationships between IPCP and patient outcomes. The proposed operational indicators (Table 3) offer a starting point for future measurement development rather than a validated measurement instrument, and should be interpreted accordingly.

#### 6.1.1 Research

Further research is needed to examine IPCP as a distinct construct that can be eventually be measured, rather than continuing to infer its impact through interprofessional education or collaboration alone. While existing literature suggests promising benefits, direct evidence linking interprofessional collaborative practice to improved care outcomes remains limited and methodologically inconsistent (Zwarenstein and Bryant, 2000; Zwarenstein et al., 2009). Among the few studies available, variability in intervention design and inconsistency in findings limit the generalizability and replicability of results (Zwarenstein and Bryant, 2000).

To strengthen the evidence base, practice-focused studies that explicitly evaluate IPCP using rigorous and consistent methodologies are required (Zwarenstein and Bryant, 2000). Such work is essential to establish IPCP as an evidence-based model of care. Future inquiry is recommended to evaluate IPCP as a clearly defined, measurable, and consistently applied construct, particularly in relation to its effects on team performance, care coordination, and patient outcomes. Advancing this research agenda is critical to validating IPCP as an effective model of collaborative care and embedding it meaningfully into clinical practice.

These findings extend, rather than duplicate, existing systematic reviews of interprofessional collaboration. Cochrane reviews of interprofessional collaboration and education have consistently found that although collaborative interventions are widely implemented, their effects on measured patient and process outcomes remain inconsistent and difficult to isolate from other quality improvement efforts (Reeves et al., 2017; Zwarenstein and Bryant, 2000). Broader reviews of interprofessional collaboration similarly emphasize the importance of sharing, partnership, and interdependency without specifying how these elements are enacted moment-to-moment in clinical teams (Schot et al., 2020; Bookey-Bassett et al., 2017). Realist syntheses of team-based collaborative care echo this pattern, identifying shared decision-making and team learning cultures as mechanisms of collaboration while noting that how these mechanisms are sustained in practice is rarely made explicit (O’Donnell et al., 2025). By identifying antecedents, attributes, and consequences specific to the enactment of collaboration, the present analysis complements this literature by offering a more granular, practice-level account that future intervention and measurement studies could build upon.

#### 6.1.2 Practice

There remains a critical need to examine how IPCP is enacted in practice and how interprofessional readiness translates into clinical outcomes. High-performing interprofessional teams influence interconnected domains of research, education, and clinical care, ultimately shaping the landscape of collaborative practice (Green and Johnson, 2015). Early and sustained exposure to IPE supports interprofessional readiness and transition into practice. In the author’s interpretation, to promote effective IPCP, individualized care planning must incorporate communication, mutual trust, and a shared mental model among team members. This positions interprofessional collaborative practice as a driver of cultural transformation toward shared purpose, collective accountability, and relationally grounded care.

Communication was the most frequently identified antecedent (*n* = 39/43), while Promoting Reciprocal Relationships was the least frequently supported (*n* = 9/43) (Table 2) suggesting that while communication functions as a near-universal, well-operationalized requirement for IPCP across the literature, reciprocity in team relationships remains conceptually underdeveloped: it recurs frequently as an adjacent idea, embedded within broader discussions of trust, mutual respect, or relationship-building, but is rarely isolated and named as its own distinct mechanism. This asymmetry may also reflect a practical reality: communication is readily structured and trained through discrete institutional mechanisms such as rounds, huddles, and handoff protocols, whereas reciprocity depends on sustained relational investment that is harder to standardize, teach, or measure, and may therefore be underrepresented in the literature not because it matters less, but because it resists the kind of operational definition that generates citable, isolable findings.

To support this shift, IPCP must be explored within healthcare systems that have yet to fully adopt shared leadership and mutual ownership. As interpreted from the literature, to enhance teamwork, both nurses and physicians must prioritize effective communication, actively engaging in structured interprofessional interventions that foster collaboration. Reciprocal relationships foster psychological safety and inclusive work environments where collaboration can genuinely thrive (Edmondson and Lei, 2014; Wei, 2021). Strengths-based approaches highlight and enhance each member’s capabilities, rather than dwelling on limitations, therefore encouraging mutual respect, reinforcing positive team dynamics, and enhancing collective efficacy (Lapum et al., 2020; Wei et al., 2020). Embedding IPCP requires intentional cultivation of environments where all team members contribute as equal and synergistic collaborators. Achieving this transformation necessitates ongoing inquiry, reflective practice, and adaptation to context. At the frontline, nursing plays a central role in operationalizing IPCP, advancing inclusive, effective, and compassionate models of care.

In critical care and trauma care environments, adequate communication through a way-of-being with patients, families, and team members is essential (Lapum et al., 2020). Relational communication approaches, such as relational caring inquiry (RCI) strengthens human connectedness and promotes open, therapeutic communication across teams (Cara, 1999; Lapum et al., 2020). An RCI approach focused on understanding and mutual connection among individuals, contexts, and relationships enhances collaboration, supports person-centric priorities, and strengthens teamwork (Lapum et al., 2020). Rooted in Caring Science, RCI is a humanistic approach to communication that emphasizes authentic presence, empathy, and meaningful engagement (Cara, 1999). Aligned with this philosophy, the IPEC Core Competencies for Interprofessional Collaborative Practice underscore the importance of communicating within teams in a responsible, respectful, and compassionate manner (Interprofessional Education Collaborative, 2023). When healthcare teams prioritize caring-trusting relationships and strengths-based collaboration, IPCP moves from being an abstract, intangible ideal to a tangible, sustainable practice that holds the potential to improve healthcare delivery while enhancing patient and provider wellbecoming (Phillips, 2017).

#### 6.1.3 Policy and global context

The recognition of interprofessional collaborative practice (IPCP) in policy and healthcare systems is complex and multifaceted, requiring coordinated, system-level alignment with regional and global healthcare priorities (Girard, 2021; Green and Johnson, 2015). Embedding IPCP requires intentional cultivation of environments that support shared leadership, equitable participation, and collaborative accountability. Achieving this transformation necessitates ongoing inquiry, reflective practice, and adaptation to context. Healthcare professionals play a central role in operationalizing IPCP through collaborative leadership, shared accountability, and person-centered team functioning.

The current evidence base is disproportionately derived from North American and European contexts, highlighting a critical gap in global representation and limiting transferability across diverse healthcare systems. This underscores the need for context-sensitive implementation of IPCP that accounts for cultural, organizational, and workforce variation.

Emerging work from the Middle East and North Africa (MENA) region provides an illustrative example of both the opportunities and challenges associated with implementing IPCP within multicultural and rapidly evolving healthcare environments (El-Awaisi et al., 2017; El-Awaisi et al., 2021; Habre et al., 2023; Rethlefsen et al., 2021; Zaher et al., 2022; Zeeni et al., 2016). Across diverse international settings, healthcare systems must navigate workforce diversity, professional hierarchies, cultural influences, and variation in collaborative practice structures. Policymakers and academic leaders are urged to prioritize curriculum reforms that embed IPE throughout undergraduate and postgraduate education (Girard, 2021; Zeeni et al., 2016). Developing a collaborative practice-ready workforce through interprofessional education and integration into clinical environments is essential for sustainable implementation of IPCP.

Policy-driven adoption of interprofessional education must be supported by infrastructure that enables interprofessional socialization in practice, including joint simulations, interprofessional clinical rounds, and collaborative decision-making opportunities (Reinders and Krijnen, 2023). Without integration into practice, the impact of interprofessional education on outcomes remains limited. Effective IPCP policy therefore depends on alignment across academic institutions, healthcare organizations, and governance systems to support communication, role clarity, and collaborative practice.

Population- and culture-specific health needs necessitate the development of collaborative practice-ready teams, equipped with interprofessional identity, cultural humility, and adaptive collaborative capabilities (Reinders and Krijnen, 2023; World Health Organization, 2010). Tailoring IPCP to address region-specific priorities, such as chronic disease management, emergency response, and culturally competent care, enhances both relevance and sustainability.

While MENA provides a salient example, these challenges are globally applicable, particularly in healthcare systems characterized by workforce diversity, evolving professional roles, and increasingly complex care delivery demands. Advancing IPCP globally requires scalable, context-sensitive strategies that integrate policy, education, and practice; strengthen interprofessional socialization; and support the implementation, evaluation, and sustainability of collaborative team functioning across healthcare systems.

### 6.2 Limitations

Several limitations of this concept analysis warrant consideration. A primary challenge is the inconsistent and interchangeable use of terminology related to interprofessional collaborative practice, including interprofessional collaboration, interprofessional education, and interprofessional readiness. While this analysis focuses on IPCP, variability in terminology may have led to the exclusion of relevant literature that reflects IPCP principles but is not explicitly labeled as such, potentially narrowing the conceptual scope and limiting comprehensiveness. This risk was addressed through the deliberate inclusion of surrogate and related terms as part of Rodgers’method (see Results, Surrogate Terms and Related Terms), though some relevant literature using unrecognized terminology may nonetheless have been missed.

Furthermore, IPCP as a distinct construct remains underdefined within the literature, with many studies addressing related concepts without clearly delineating its boundaries. This results in conceptual overlap and dilution, limiting the precision of both theoretical development and practical application. Future concept analyses would benefit from more explicit criteria to differentiate IPCP from adjacent constructs.

This analysis is inherently time-bound, reflecting the body of literature available at the time of review. Given the evolving nature of interprofessional teams, healthcare systems, and care delivery models, the conceptualization of IPCP is likely to continue developing across contexts. This temporality limits generalizability, particularly in diverse cultural and organizational settings. A targeted update search was conducted in 2025 to reduce this limitation, though the analysis remains bound to literature available at the time of review.

Finally, the limited number of studies explicitly examining IPCP highlights an important gap in the literature, particularly regarding the roles of patients and families as active participants in interprofessional teams. This omission is notable given the centrality of person-centered care and patient partnership to IPCP. As an integrative concept analysis, the study did not include formal methodological quality appraisal or risk-of-bias assessment; therefore, the findings reflect the conceptual representations within the included literature rather than the methodological strength of individual studies. Future research should prioritize the integration of patient and family perspectives and employ methodological approaches capable of capturing IPCP as an enacted and observable form of collaborative team functioning within real-world clinical settings.

## 7 Conclusion

Interprofessional collaborative practice is a complex and evolving concept shaped by disciplinary perspectives and the practical realities of healthcare delivery. Consistent with Rodgers’ evolutionary method, IPCP continues to be refined across time, culture, and practice environments (Bookey-Bassett et al., 2017; Rodgers and Knafl, 2000). This concept analysis affirms that IPCP is distinct from related constructs such as interprofessional education and interprofessional collaboration: whereas those constructs address understanding of, and readiness for, collaboration, IPCP centers on how collaborative team functioning is enacted in practice.

Based on these findings, IPCP is defined as collaborative team functioning enacted through shared decision-making, reciprocal relationships, role clarity, and coordinated communication among healthcare professionals, patients, and families to achieve safe, effective, and person-centered care. By identifying its antecedents, attributes, consequences, and exemplars, this analysis offers a foundation for future work to operationalize, implement, and evaluate collaborative team functioning in clinical guidelines, team-based interventions, and healthcare practice more broadly. Accordingly, IPCP is advanced as a researchable and actionable construct with strong potential to inform healthcare practice, policy, future measurement development, and interprofessional care frameworks.

## Supplementary material

Supplementary Table 1 Literature Matrix Codebook. Coding framework for the evolutionary concept analysis, comprising operational definitions and criteria for each antecedent, attribute, and consequence as these evolved iteratively through the coding process, paired with the literature matrix documenting notes and supporting quotes drawn from each source.

## Appendix

### Search strings and database search strategies.

This appendix provides the full search strategies used across the three databases searched for this concept analysis (PubMed, Embase, and CINAHL), reported in accordance with PRISMA-S (Rethlefsen et al., 2021) guidance for literature search transparency.

Deduplication across databases was performed using EndNote 21 (167 duplicates removed). See Figure 1 for the full search, screening, and selection flow diagram.

**PubMed (MEDLINE)**

**Table:**

Database:	MEDLINE (PubMed)
**Search period:**	January 2014–December 2024, with a targeted update search conducted in 2025
**Search string:**	#1 “interprofessional collaborative practice”[tiab] OR “interprofessional collaboration”[tiab] OR “interprofessional relations”[tiab] OR “Interprofessional Relations”[MeSH] OR “interdisciplinary collaboration”[tiab] OR “collaborative practice”[tiab] OR teamwork[tiab] OR “Patient Care Team”[MeSH] #2 “acute care”[tiab] OR nursing[tiab] OR caring[tiab] OR “team-based care”[tiab] OR “healthcare team*”[tiab] OR “interprofessional team*”[tiab] #3 #1 AND #2
**Limits:**	English language; publication dates 2014/01/01–2024/12/31
**Records retrieved:**	*n* = 197

**Embase**

**Table:**

Database:	CINAHL Complete (EBSCOhost)
**Search period:**	January 2014–December 2024, with a targeted update search conducted in 2025
**Search string:**	S1 (MH “Interprofessional Relations”) OR (MH “Patient Care Team”) OR “interprofessional collaborative practice” OR “interprofessional collaboration” OR “collaborative practice” OR teamwork S2 (MH “Acute Care”) OR nursing OR caring OR “team-based care” OR “healthcare team*” OR “interprofessional team*” S3 S1 AND S2
**Limits:**	English language; Publication date: 1 January 2014–31 December 2024.
**Records retrieved:**	*n* = 81

**CINAHL**

**Table:**

Database:	Embase.com
**Search period:**	January 2014–December 2024, with a targeted update search conducted in 2025
**Search string:**	#1 “interprofessional collaboration”/exp OR “interprofessional relations”/exp OR “patient care team”/exp OR “interprofessional collaborative practice”:ti,ab OR “collaborative practice”:ti,ab OR teamwork:ti,ab #2 “acute care”/exp OR nursing:ti,ab OR caring:ti,ab OR “team based care”:ti,ab OR “healthcare team*”:ti,ab OR “interprofessional team*”:ti,ab #3 #1 AND #2
**Limits:**	English language ([english]/lim); publication years 2014–2024 ([2014–2024]/py)
**Records retrieved:**	*n* = 106

Search strategies are reported here as executed for the primary search (2014–2024); the 2025 search update applied the same concept structure with the date limit adjusted accordingly. Field tags and syntax are platform-specific and are not directly interchangeable across databases.
